# Combining the Grisotti Flap With a Secondary Dermoglandular Pedicle for Partial Breast Reconstruction Following Contiguous Central-Inferior Segment Breast Cancer Excision

**DOI:** 10.1080/23320885.2021.2008801

**Published:** 2021-12-01

**Authors:** Charles M. Malata, Isabel Jia Le See, Fawz Kazzazi, Parto Forouhi, Bruno Di Pace

**Affiliations:** a Department of Plastic and Reconstructive Surgery, Addenbrooke’s Hospital, Cambridge University Hospitals NHS Foundation Trust, Cambridge, UK; b Anglia Ruskin School of Medicine, Anglia Ruskin University, Cambridge, UK; c Cambridge Breast Unit, Addenbrooke’s Hospital, Cambridge University Hospitals NHS Foundation Trust, Cambridge, UK; d Faculty of Medical and Health Sciences, University of Auckland, Auckland, New Zealand; e Clinical School of Medicine, University of Cambridge, Cambridge, UK; f The Royal London Hospital, Barts NHS Health Trust, London, UK; g Department of Medicine, Surgery and Dentistry “Scuola Medica Salernitana”, PhD School of Translational Medicine of Development and Active Aging, University of Salerno, Salerno, Italy

**Keywords:** Grisotti flap, partial breast reconstruction, central breast excision, breast-conserving surgery, oncoplastic surgery, secondary dermoglandular pedicles

## Abstract

A 61-year-old patient (38DD) with multifocal invasive ductal carcinomas requested breast-conserving surgery. An innovative two pedicle combination using a laterally-based Grisotti flap and an inferomedially-based secondary pedicle was designed to reconstruct a combined central breast (NAC included) and inferior segment resection defect. Satisfactory cosmesis with clear resection margins was achieved.

## Introduction

A key objective in partial breast reconstruction is achieving radical oncological resection whilst aiming for the best aesthetic outcome by employing oncoplastic surgical techniques [1–4]. However, when faced with centrally located breast cancers, surgeons often opt for mastectomy rather than breast-conserving surgery (BCS) as the nipple areolar complex (more often than not) has to be sacrificed thereby negating a key purpose of BCS. This decision is often due to the inherent difficulty in reconstructing central breast defects [4].

The Grisotti dermoglandular rotation-advancement flap and its modifications [5–9], is an established method for partial breast reconstruction after the removal of centrally located tumours where the nipple areolar complex (NAC) is sacrificed [10]. It is particularly useful for moderate to large sized breasts [11]. However, the flap is insufficient for reconstructing larger and wider defects that extend into an adjacent area.

Therefore, we designed a technical modification which utilises two dermoglandular pedicles: a laterally-based Grisotti flap and an inferomedially-based secondary pedicle to reconstruct a combined central and inferior segment defect leading to satisfactory cosmetic results with clear resection margins. A simultaneous contralateral balancing breast reduction was performed for symmetry.

## Case

The modification design was applied to a 61-year-old asymptomatic patient (bra cup size 38DD) who was recalled for a right breast abnormality discovered on routine screening. She was found to have two separate foci of Grade 1 invasive ductal carcinoma that were 4 cm apart in the upper part of her right breast.

**Figure 1.**
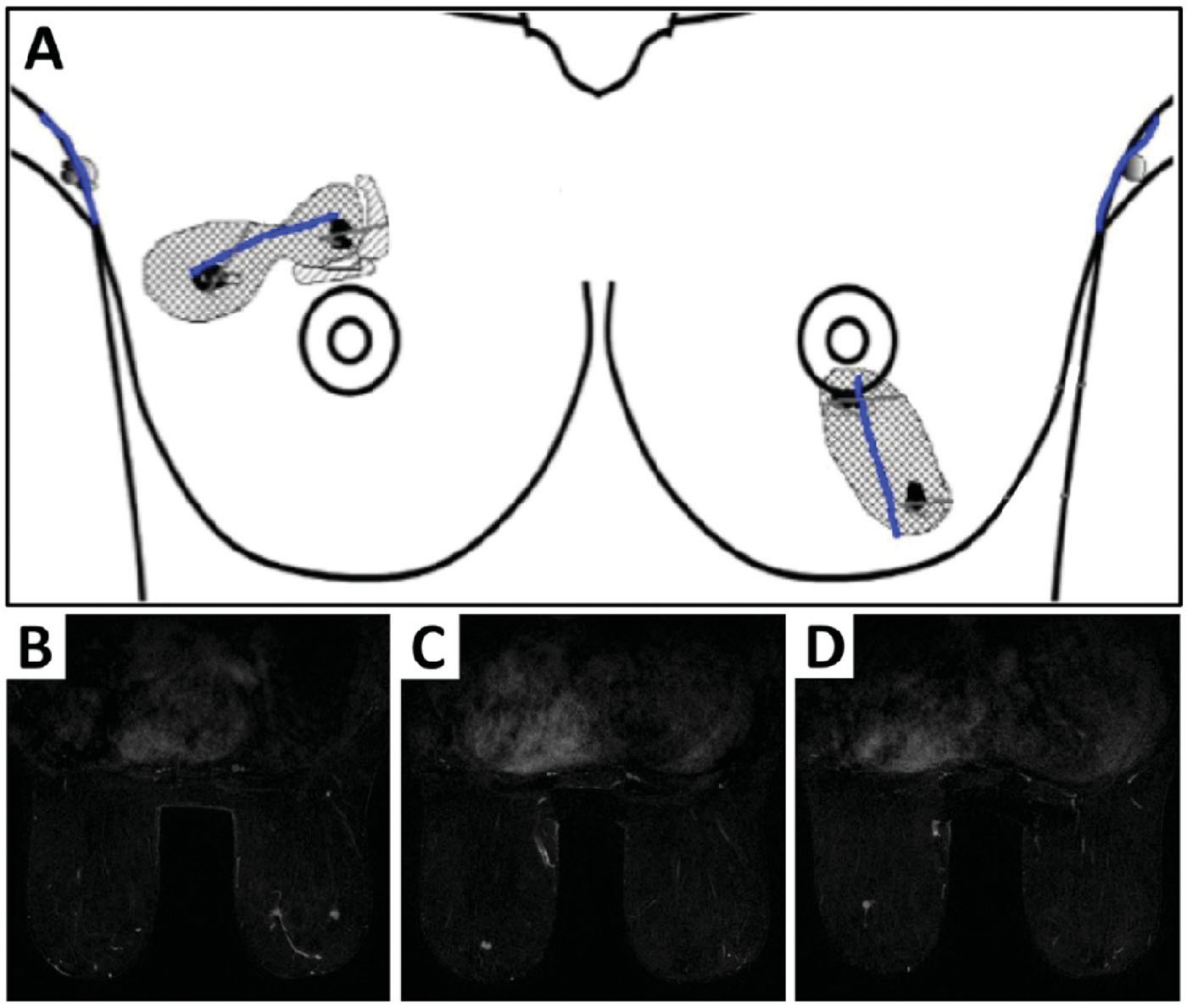
Illustration (A) showing two foci of Grade 1 invasive ductal carcinomas (4 cm apart) in the right breast and two foci of Grade 1 invasive ductal carcinomas (5 cm apart) in the inferior-central and inferior part of the left breast. Bilateral breast MRI with contrast (B) showing an irregular enhancing lesion in the upper outer quadrant measuring 12 × 8 × 13 mm and a second malignancy, measuring 9 × 6 × 11 mm in the upper medial aspect of the right breast at the same level as lesion 1. The left breast (C) shows a 7 mm irregular enhancing lesion at the 6 o’clock position and (D) a second lesion measuring 5 mm located inferolateral to this.

She expressed a strong preference to pursue breast-conserving surgery (BCS) and a magnetic resonance imaging (MRI) was performed to assess the full extent of the disease. No additional disease was discovered in the right breast, but two abnormal areas in the inferior-central and inferior part of her left breast 5 cm apart were revealed (Figure 1). These two lesions were subsequently confirmed to be foci of Grade 1 invasive cancers on biopsy.

The patient underwent bilateral BCS, including sentinel node biopsies, using a direct segmental excision on the right (72 g), and a vertical inferior mammoplasty on the left (42 g). Cavities were closed using local tissue displacement. Histology revealed complete excision of the right tumour complex (10 and 11 mm invasive foci associated with intermediate grade ductal carcinoma *in situ* [DCIS]). On the left side, although the invasive foci were excised, these were associated with further 59 mm of low-grade DCIS that was close to both the superior (nipple) and inferior margins.

The patient chose to pursue a second attempt at breast conservation on the left. Given the segmental distribution of the disease and the extension of DCIS to the nipple, she was offered the excision of the entire lower segment of the breast to include the NAC. This was to be performed along with balancing reduction on the right side to preserve breast symmetry.

## Surgical technique

### Re-wide local excision of left breast

A direct radial curvilinear incision was made incorporating the NAC. Approximately 1–2 cm of skin was released around the edge of the incision and the tumour was excised anteriorly to the skin and posteriorly to the pectoral fascia with a diathermy resection. The resected specimen weighed 128 g.

### Left breast reconstruction

**Figure 2.**
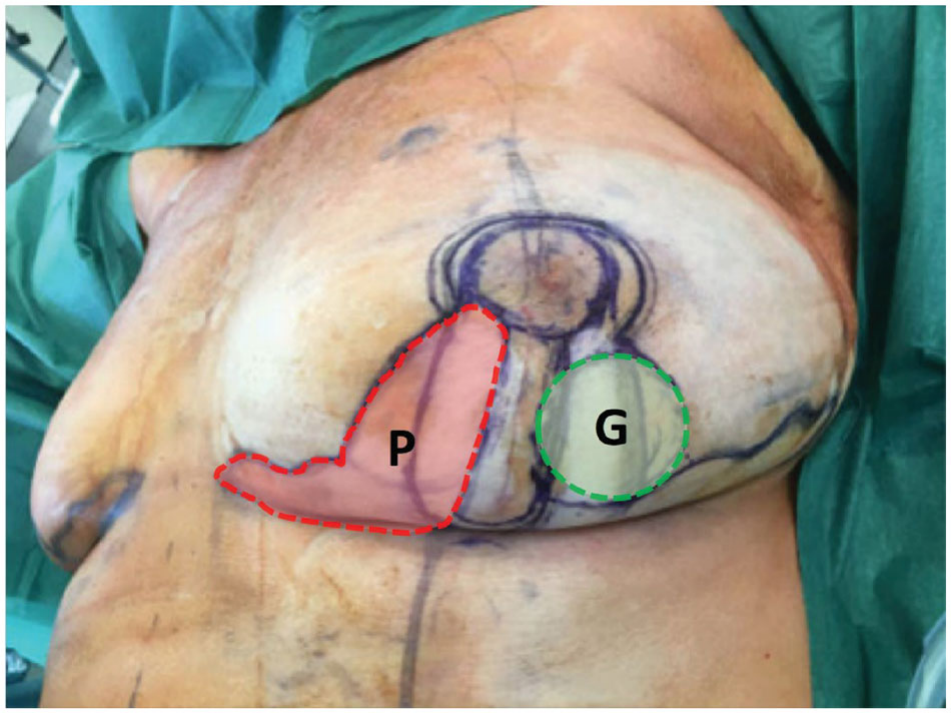
Intraoperative surgical markings on the left index breast outlining a round neoareola (shaded green, marked G) of the inferolaterally-based Grisotti flap and the location of the inferomedial secondary pedicle with its overlying skin (shaded red, marked P) prior to de-epithelialisation. The Wise pattern (deep black markings) is superimposed on these markings.

A skewed Wise pattern incorporating a Grisotti flap and a secondary pedicle was designed (Figure 2).

**Figure 3.**
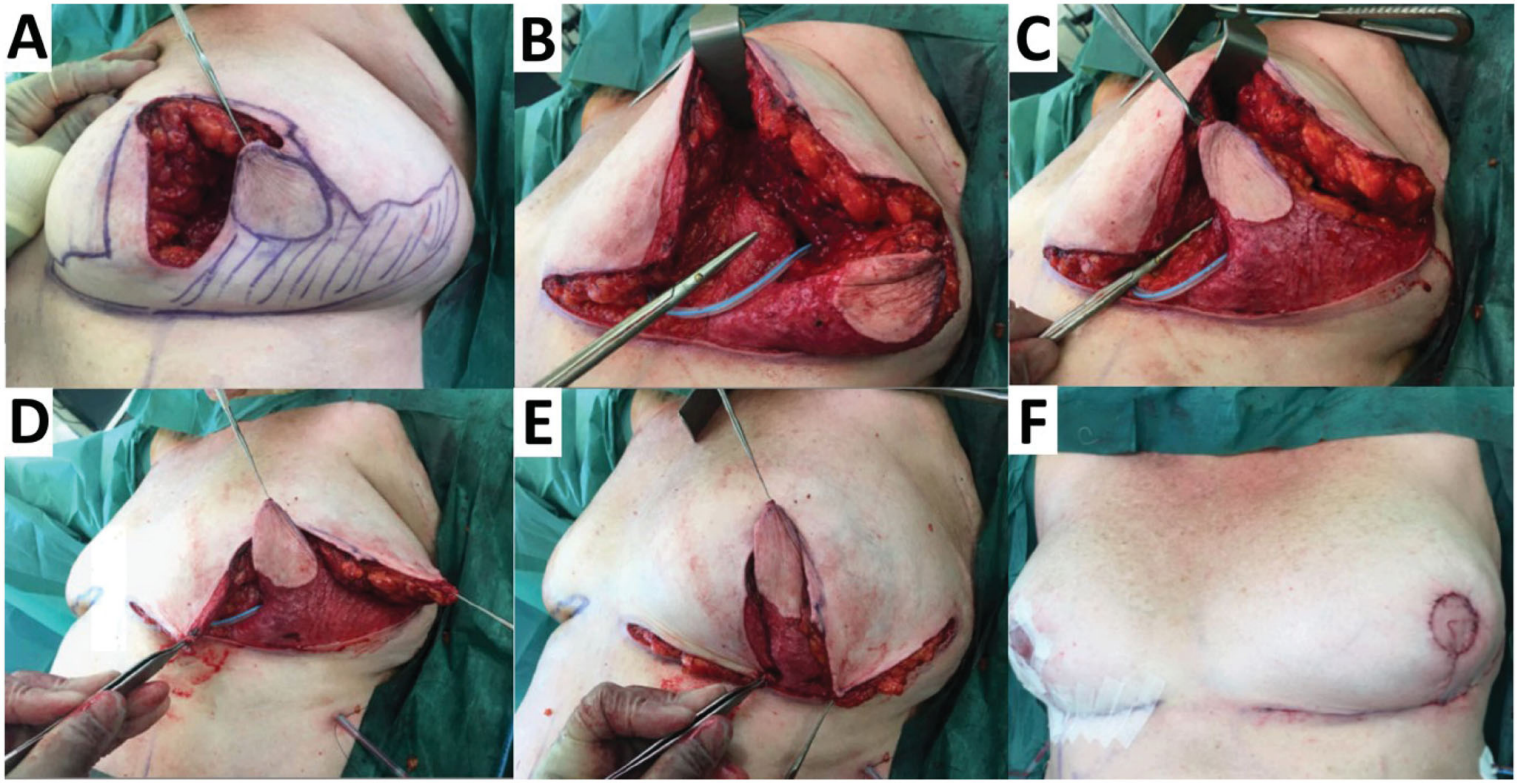
(A) Following central and inferior segment excision on left breast, a circular skin paddle (neo-areola) on the laterally-based Grisotti flap is marked. The shaded zone is the part of the Grisotti flap that will be de-epithelialised. The part of the breast medial to the inferior segmental defect inside the Wise pattern markings will be totally de-epithelialised and constituted the secondary pedicle. (B) The inferomedially-based secondary pedicle (underneath the scissors) is transposed and advanced into the deep part of the central defect which is about to be buried by suturing it to the pectoral fascia. (C) After fixation of secondary pedicle into the central defect, the Grisotti flap is advanced into the defect on top of/superficial to the secondary pedicle. (D) The Wise pattern ‘wings’ are being prepared to be approximated superficially to the de-epithelialised part of the Grisotti flap. (E) Both flaps (Grisotti & secondary pedicle), which are designed to fill central and inferior cavity defect, are now in place prior to closure. (F) Completion of the right superomedial pedicle Wise pattern breast reduction and breast-conserving surgery on the left breast, showing reasonable on-table symmetry.

Following central and inferior segmental breast excision, the defect was reconstructed with a laterally-based Grisotti flap, which was combined with an inferomedially-based secondary dermoglandular pedicle (Figure 3). The Grisotti flap based laterally was employed as the positive margin abutted the medial side of the previous vertical scar and needed further excision on the medial side (Figure 3(A)).

The laterally-based Grisotti flap was raised with back cuts into the tight dermis at its base. As the flap was not thick enough to fill the depth of the central excision, an inferomedially-based secondary dermoglandular pedicle (that was intended to be totally buried) was then raised (Figure 3(B)). This secondary pedicle was completely de-epithelialised and transposed as a second flap into the deep part of the central defect underneath the Grisotti flap (Figure 3(C)). The secondary dermoglandular flap was secured to the underlying pectoral fascia and the surrounding breast tissue. The Grisotti flap was then advanced and secured into the superficial part of the defect (Figure 3(D)). The Grisotti skin paddle was then trimmed to fit the planned neo-areola (at the site of excision of the NAC) (Figure 3(E)). Its inferior dermis was also partially divided to enable better flap inset.

A suction drain was inserted, and the wounds closed in three layers were then dressed with steristrips, gauze and supportive microfoam tape.

### Right balancing reduction mammoplasty

A contralateral balancing Wise pattern breast reduction (252 g) using a superomedial nipple pedicle was carried out to improve symmetry (Figure 3(F)).

## Post-operative outcome

**Figure 4.**
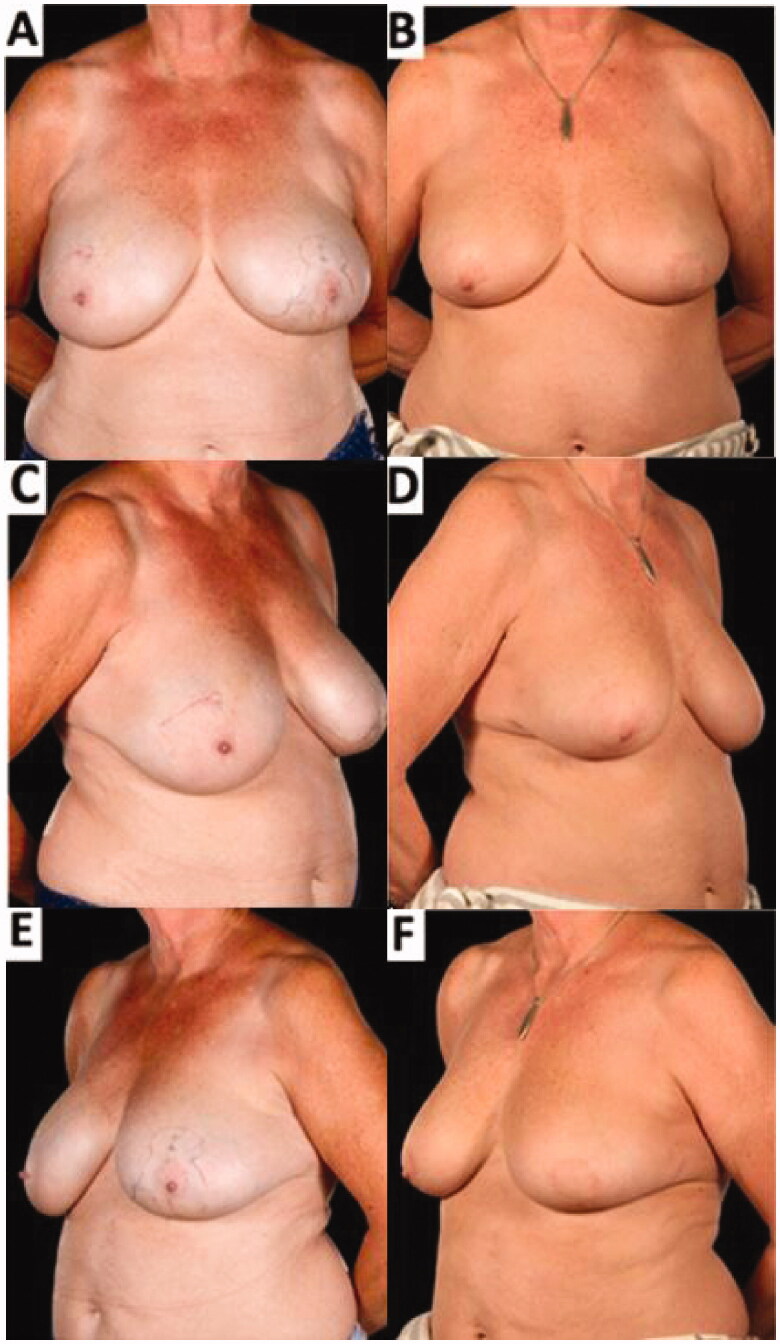
Pre-operative (A,C,E) and 8-months post-operative (B,D,F) appearances of a 61-year-old patient who underwent breast-conserving surgery comprising a redo-wide local excision (central and inferior segmental resection including the NAC) reconstructed with a laterally-based Grisotti flap and an inferiorly-based secondary dermoglandular pedicle and simultaneous contralateral symmetrising breast reduction – 6 months after completion of adjuvant radiotherapy. The improved breast symmetry was made possible by the complex oncoplastic techniques. The patient declined formal nipple reconstruction preferring intermittent use of a prosthetic nipple. There was no residual visible skin reaction from the radiotherapy treatment.

Histology of the resected specimen showed no abnormality in the right breast reduction specimens. On the left, there was a further 65 mm of low-grade DCIS which had been completely excised with wide clear margins. Post-operatively, she received adjuvant bilateral whole breast radiotherapy and 5 year adjuvant endocrine treatment. The patient reported that she was very satisfied with the cosmetic appearance of her breasts and has since declined nipple reconstruction (Figure 4). She remains symptom and disease free at 1-year follow-up and will undergo annual screening according to breast cancer patient protocol.

## Discussion

Centrally located breast cancers often lead to the most unfavourable cosmetic results of all other quadrantectomies, and indeed tend to lead surgeons to opt for mastectomy rather than breast conversation surgery (BCS), thereby providing impetus for continually improved oncoplastic techniques [12–14].

Due to the multifocal invasive ductal carcinomas in the inferior-central and inferior parts of the patient’s left breast, the Grisotti flap alone was insufficient in thickness to fill the depth of the central excision. Therefore, the advantages of combining the Grisotti flap with an additional buried secondary pedicle made it possible to reconstruct a larger defect extending into an adjacent area that could not be safely or easily reconstructed with one flap. This combination achieved adequate tumour margins as well as an enhancement of the breast shape with satisfactory aesthetic results (Figure 4).

The main indication for employing this combination is large-breasted patients with central and inferior tumour defects opting for BCS, as with our presented case. However, there is no literature describing how reconstruction can be carried out in patients with multifocal cancers, and specifically reconstructing a combined central nipple-sacrificing defect with a contiguous lower quadrant tumour defect. This combination adds to the repertoire of more commonly used oncoplastic techniques in partial breast reconstruction. To our knowledge, this is the first report of this innovative combination of a Grisotti flap and a secondary ‘buried’ dermoglandular pedicle.
